# Not Your Typical Pneumonia: A Rare Case of Delftia acidovorans Pneumonia With Concurrent Malignant Squamous Cell Carcinoma

**DOI:** 10.7759/cureus.79796

**Published:** 2025-02-27

**Authors:** Kyle S Gordon, Diego Marin

**Affiliations:** 1 Internal Medicine, Florida Atlantic University Charles E. Schmidt College of Medicine, Boca Raton Regional Hospital, Boca Raton, USA; 2 Pulmonary and Critical Care, Boca Raton Regional Hospital, Boca Raton, USA

**Keywords:** atypical pneumonia, delftia acidovorans, infections in immunocompromised, infections in malignancy, pneumonia, squamous cell lung carcinoma, stenotrophomonas maltophilia infection

## Abstract

*Delftia acidovorans* (*D. acidovorans*) is classified as a Gram-negative, aerobic non-fermenting bacillus that is commonly found in outdoor environmental elements, such as soil and water. In normal circumstances, it is considered a non-pathological environmental organism, rarely implicated in clinical settings with a low incidence of patient infection. *D. acidovorans* infections, including pneumonia and sepsis, are often associated with increased susceptibility to secondary infections from other Gram-negative bacteria, especially in patients with solid or hematologic malignancies. Henceforth, this case report presents a case of a stroke patient, found to have an obstructive metastatic squamous cell carcinoma of the lung complicated with *D. acidovorans* pneumonia. The report sheds light in regards to how rare, unassuming pathogens can have significant clinical significance and limited treatment modalities in immunocompromised patients.

## Introduction

*Delftia acidovorans *is a Gram-negative, aerobic, non-fermenting bacilli found in environmental sources such as soil and water. Infections by this organism are exceedingly rare and oftentimes in those with weakened immune systems and associated comorbid conditions. Oftentimes, infections are polymicrobial, which further adds to diagnostic challenges. There is a known association with *Stenotrophomonas maltophilia, *with both exhibiting multiple intrinsic drug resistances leading to difficult-to-manage infections. Both organisms have gained clinical significance, particularly in immunocompromised individuals, despite their relatively low incidence. Infections can occur in the respiratory tract, bloodstream, endocardium, ocular lens, or in the peritoneal cavity. *D. acidovorans *infections, including pneumonia and sepsis, are often associated with increased susceptibility to secondary infections from other Gram-negative bacteria, especially in patients with solid or hematologic malignancies. Previous retrospective cohort studies found that, in 59 patients with *D. acidovorans *infections, 15 (25%) died within 365 days after infection. Interestingly, a study also revealed that 97% of patients were associated with a least one comorbidity, including 42% with solid or hematologic malignancies [1]. However, *D. acidovorans* has demonstrated concern for antibiotic resistance to aminoglycosides and select cephalosporins [2,3]. Infections with *D. acidorvans* were noted in one group to be associated with *Pseudomonas* and *S. maltophilia *in 70% of cases [1,4]. Immunocompromised patients with polymicrobial infections with *D. acidovorans *in hindsight are likely at risk for rapid decompensation. Henceforth, we report a rare case of severe *D. acidovorans* pneumonia complicated by a concurrent *S. maltophilia* infection in a patient with squamous cell carcinoma. This case highlights the potential clinical impact of these uncommon pathogens and underscores the limited literature on treatment options for critically ill, immunocompromised patients infected with these organisms.

## Case presentation

A 78-year-old female, with a history of tobacco abuse, stroke, peripheral artery disease, type 2 diabetes, and hypertension, presented to the nearest hospital with altered mental status and weakness. STAT CT head revealed an acute left middle cerebral artery (MCA) stroke, and the patient underwent emergent thrombectomy with interventional radiology. Despite a successful thrombectomy for the stroke, her mental status slowly declined, she became more obtunded, and she developed respiratory failure due to poor management of oral secretions. Her respiratory failure leads to intubation within 24 hours due to her worsening mental status baseline. While in the neuro ICU, a physical exam revealed right neck swelling and palpable right cervical lymphadenopathy. CT neck and chest were obtained during this interval and revealed extensive cervical, mediastinal, and hilar lymphadenopathy, a loculated right pleural effusion, and a discrete right perihilar lung mass. Upon further questioning with the family, the patient was aware of a right lung mass previously found on a low-dose CT chest but had not sought follow-up for an unclear amount of time. Given the location of the right lung mass and the effusion, it was not believed that these were the primary drivers for her respiratory failure at that time but rather her cerebrovascular accident. Further workup was deferred at that time due to her neurologic issues. Due to their inability to participate in weaning trials, she underwent tracheostomy and percutaneous endoscopic gastrostomy placement 10 days after the initial presentation. Given her clinical stability on day 16, she was transferred to a long-term acute care facility for further care.

**Figure 1:**
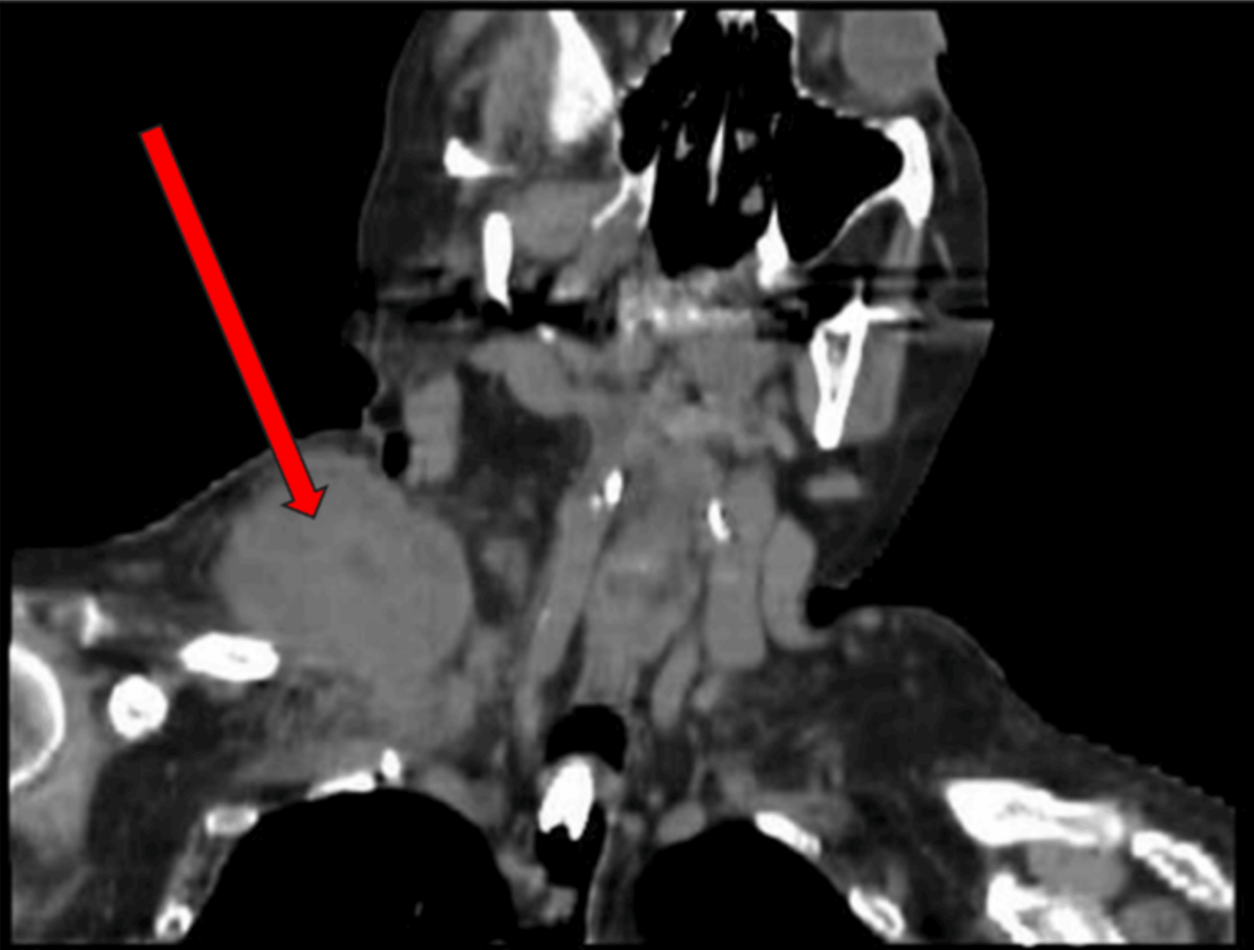
CT neck without contrast of right supraclavicular mass The red arrow depicts a significant interval increase of the right supraclavicular mass, most concerning for a primary neoplasm or metastatic disease, now measuring 7.1 x 6.1 x 8.0 cm. The mass extends below the clavicle approximately 3 cm. The mass also appears to invade the posterior aspect of the right sternocleidomastoid muscle.

**Figure 2:**
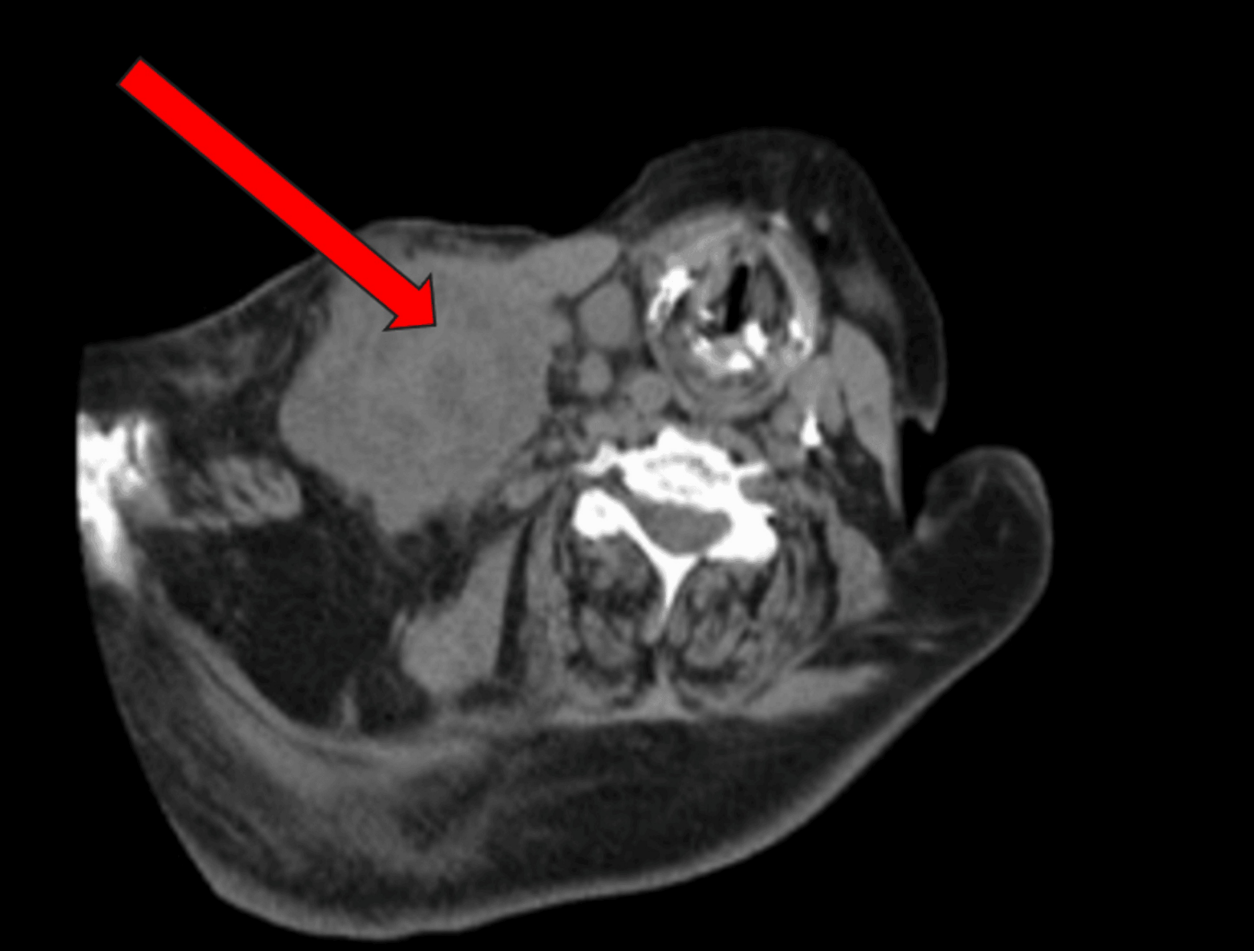
CT neck without contrast The red arrow indicates the interval enlargement of the right paraclavicular neck mass, which extends into the infraclavicular region adjacent to the right subclavian vein.

**Figure 3:**
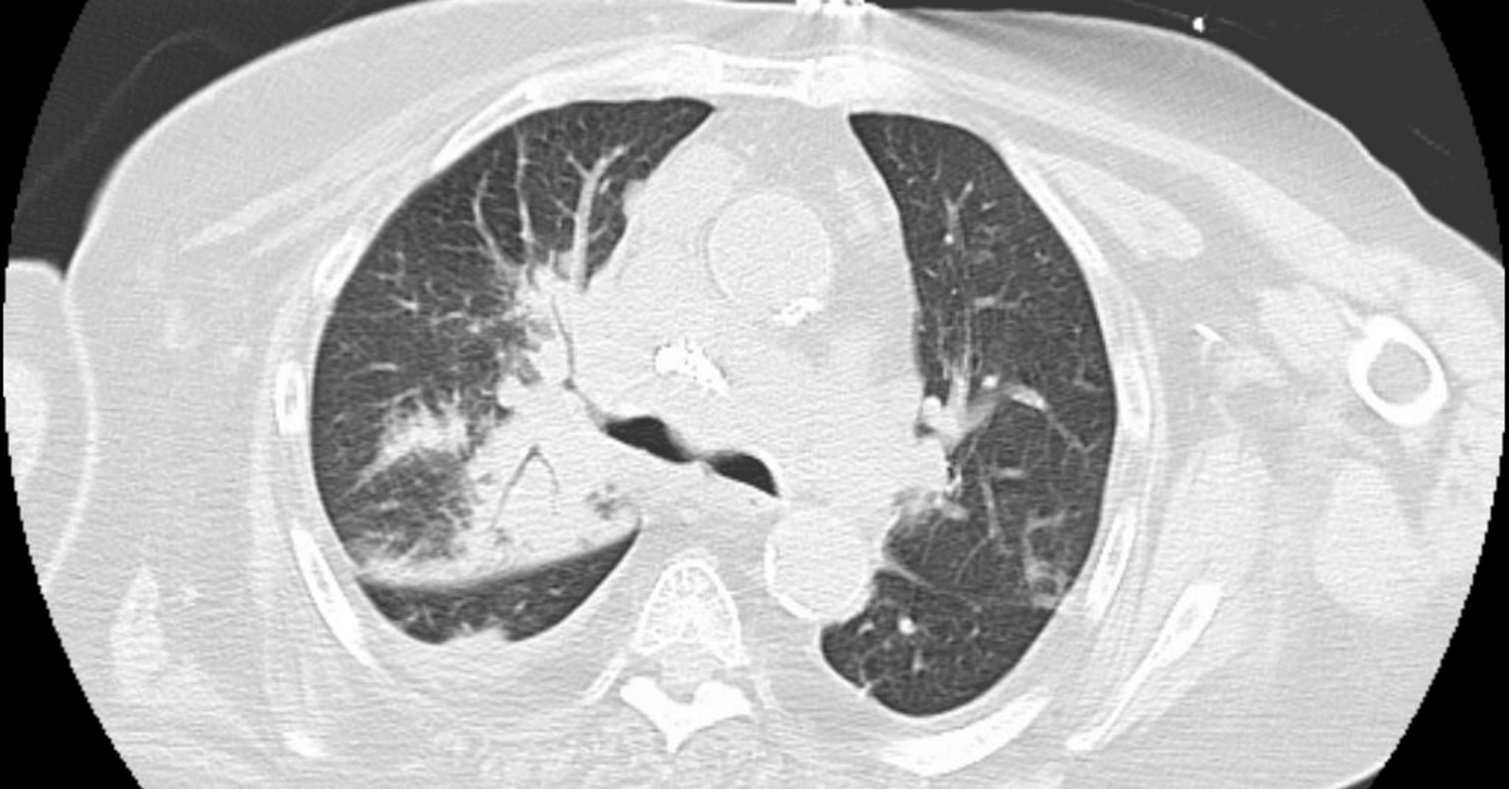
CT chest with a lung view Right hilar mass and adjacent consolidation concerning for a combination of pneumonia and interstitial edema due to lymphatic obstruction (worse than prior). Findings are consistent with malignancy and metastatic disease.

A diagnostic supraclavicular biopsy was performed at the long-term acute care facility shortly after arrival and confirmed metastatic squamous cell carcinoma of skin/cervix origin, with immunohistochemistry being positive for P63 and CK7. Given her frailty and poor functional status, oncology was unable to recommend immediate therapies. Two months into her stay, she began to develop rhonchi on exam and difficulty with further weaning. Respiratory therapists noted a slowly increasing fraction of inspired oxygen (FiO_2_) requirements and thick sputum upon suctioning. A repeat CT chest was obtained. Figure 1 presents an interval increase of the right supraclavicular mass. Figure 2 provides a different perspective of the right supraclavicular neck mass, which extends into the infraclavicular region adjacent to the right subclavian vein. Figure 3 highlights the associated right hilar mass and new areas of consolidation and bronchograms, consistent with superimposed pneumonia. Sputum cultures were obtained using tracheal aspirate and grew multidrug-resistant *Escherichia coli, *which was managed by infectious disease with meropenem. While she originally had resolution of that episode and a return to slow but progressive weaning attempts from the ventilator, a second episode of ventilator-associated pneumonia occurred shortly thereafter, about a month later. This time cultures obtained using tracheal aspirate grew* D. acidovorans and S. maltophilia, *both resistant to gentamicin. Despite management for multiple days with levofloxacin and minocycline by infectious disease, her condition continued to deteriorate and she expired.

## Discussion

*D. acidovorans* is classified as a Gram-negative, aerobic, non-fermenting bacillus that is commonly found in outdoor environmental elements, such as soil and water. Typically viewed as an uncommon pathogen for infections in the immunocompetent, it has gained notoriety for rare opportunistic infections in immunocompromised patients with malignancy or significant comorbidities, such as end-stage renal disease. Current literature on *D. acidovorans *denotes few reports of infections in immunocompromised individuals with few protocols for management strategies.

One instance of *D. acidovorans* peritonitis infection in a patient with end-stage renal disease undergoing intraperitoneal hemodialysis reported resistance to both aminoglycosides and colomycin, leading to treatment with ceftazidime and oral ciprofloxacin [3]. A peritoneal catheter was subsequently removed because of concern for refectory infection. In this case, the patient had a mild clinical course, responding well to antibiotics.

Another interesting case published in 2019 from Cali, Colombia, demonstrated the first documented case of *D. acidovorans* pneumonia-causing cavitary lung lesions in patients on immunosuppressive therapy for thrombocytopenic purpura for five years [5]. The patient subsequently underwent empirical treatment with intravenous piperacillin-tazobactam and oral clarithromycin. The presence of cavitary lesions suggests a more aggressive, advanced infection or possible prior granulomatous infectious process, but further data are needed to elucidate whether *D. acidovorans *infection can directly lead to cavitary lesions. Interestingly, this case did not reveal a polymicrobial co-infection with organisms such as pseudomonas or *S. maltophilia*, as demonstrated in our case, as the limited current literature indicates that *D. acidovorans *infections are primarily polymicrobial [1].

Finally, a retrospective cohort study evaluated patients who were diagnosed with *D.acidovorans *and found a high prevalence of multiple comorbidities, especially preexisting malignancies [2]. This report revealed that treatment outcomes showed a mortality rate of 11.5%, mainly in patients with malignancy and advanced age similar to our patient's case. In addition, this retrospective study found that approximately 76% of *D. acidovorans* infection patients had polymicrobial infections. Interestingly, they found that individuals who received antibiotics within the past three months were at increased risk of *D. acidovorans *infection. These results correlate with our patient, since the patient previously received antibiotics due to initial sputum cultures testing positive for multi-drug-resistant *E. coli*, before being diagnosed with *D. acidovorans *approximately 10 days later.

## Conclusions

Infections with *D. acidovorans* are a rare occurrence primarily observed in immunocompromised individuals, specifically with metastatic malignancy, as in our patient or advanced cardiovascular disease. It has been found that infections with *D. acidovorans* are often polymicrobial, with a high resistance rate to aminoglycosides. Individual cases highlight response and improved outcomes when treated with antibiotics with anti-pseudomonas activity. Both the polymicrobial nature and resistance to antibiotics make treatment decisions complex, and prolonged regimens are sometimes necessary. Collaborative efforts with infectious disease specialists are pivotal in the management of these rare infections. The noted high mortality rate of 25% within one year or *D. acidovorans* infections are likely due to a multitude of the aforementioned factors, particularly the presence of multiple comorbidities. Given the advancing age and morbidity of multiple populations, *D. acidovorans *may be an emerging pathogen with little medical literature available to guide management.
